# Target-Controlled Sedation with Propofol Infusion for PEG-J Placement in Advanced Parkinson’s Disease: A Prospective Observational Study on Safety and Feasibility

**DOI:** 10.3390/life15030492

**Published:** 2025-03-18

**Authors:** Janos Szederjesi, Irina Săplăcan, Alexandra Lazăr, Matild Keresztes, Georgiana-Mihaela Muller-Șerban, Jozsef Attila Szasz, Bianca Liana Grigorescu

**Affiliations:** 1 Department of Intensive Care, George Emil Palade University of Medicine, Pharmacy, Science and Technology of Târgu-Mureș, 540139 Târgu Mureș, Romania; yangzi37@gmail.com (J.S.); alexandra.lazar@umfst.ro (A.L.); keresztesmatild@yahoo.com (M.K.); georgiana.serban94@yahoo.com (G.-M.M.-Ș.); biancagrigorescu20@yahoo.com (B.L.G.); 2 2nd Clinic of Neurology, Târgu Mures, County Emergency Clinical Hospital, George Emil Palade University of Medicine, Pharmacy, Science and Technology of Târgu-Mureș, 540139 Târgu Mureș, Romania; szaszneuro@yahoo.com

**Keywords:** Parkinson disease, TCI, anesthesia protocol, levodopa–entacapone–carbidopa intestinal gel

## Abstract

Introduction: The management of sedation during percutaneous endoscopic gastrojejunostomy (PEG-J) placement in patients with advanced Parkinson’s disease (PD) is challenging due to the complex interactions between PD treatment, anesthetic agents, and the disease’s motor and non-motor symptoms. This study evaluates the effectiveness and safety of a target-controlled infusion (TCI) propofol protocol in the context of PEG-J placement in advanced PD patients. Materials and Methods: This prospective study included 169 patients diagnosed with advanced Parkinson’s disease (Hoehn and Yahr stages 4 and 5) who underwent PEG-J placement at Târgu Mureș County Emergency Clinical Hospital, Romania. Sedation was induced and maintained using TCI propofol, with additional benzodiazepines and short-acting opioids, while muscle relaxants were not used. Procedural success rates and adverse outcomes were assessed for 30 days post-procedure. Results: The sedation protocol demonstrated a high procedural success rate. No deaths were reported within 30 days post-procedure. Conclusion: This study highlights the feasibility and clinical applicability of a TCI propofol protocol for PEG-J placement in patients with advanced PD (stages 4 and 5). While no deaths were recorded within the 30-day follow-up, the sample size is insufficient to draw definitive conclusions regarding long-term safety.

## 1. Introduction

Parkinson’s disease (PD) is the most common neurodegenerative condition affecting movement. The estimated prevalence of PD is 0.3% in the general population of industrialized countries. Among people older than 60 years, the prevalence increases to 1.0%, and for those older than 80 years, it further rises to 3.0%. The estimated prevalence and incidence rates of PD in Europe range from 65 to 12,500 cases per 100,000 people. In Romania, as of 2018, it was estimated that more than 73,000 individuals were living with PD [1]. Our center is one of the five specialized centers in Romania for the treatment of PD where Levodopa therapy can be initiated and managed. Among these, our institution has the largest patient cohort, making it the most experienced center in the country in managing advanced PD and PEG-J placement.

Levodopa (LD) remains the gold standard for symptom relief, but long-term use leads to motor complications affecting quality of life. Dopamine agonists can be used alone or with LD, especially in the early stages [2]. Enteral LD infusion bypasses gastroparesis-related absorption issues, ensuring more stable drug delivery [3].

Levodopa–carbidopa intestinal gel (LCIG) intra-jejunal infusion is a proven long-term treatment for advanced Parkinson’s disease (APD) with severe motor fluctuations, with or without dyskinesias [4]. Its 16 h continuous administration effectively improves motor and non-motor symptoms, as confirmed by multiple studies [5]. Daytime and nighttime administration are options when medically justified.

### Perioperative Risk

Patients with PD face an increased risk of postoperative complications. Common issues include pulmonary aspiration, urinary tract infections, wound infections, respiratory insufficiency, hypotension, delirium, and pressure sores. The elevated complication rate in PD patients is attributed to multiple factors, including worsening Parkinson-related motor and non-motor symptoms, bulbar dysfunction, immobility and fall risk, challenges in managing complex medication regimens, and advanced age accompanied by multiple comorbidities [6].

There is no universally safe anesthetic regimen for PD patients, and available safety data is primarily based on case reports or series. When appropriate, regional or central neuraxial block provides several advantages. However, intense stiffness and tremors in body areas not affected by anesthesia can complicate placement and monitoring [6,7].

General anesthesia may mitigate this challenge by temporarily eliminating Parkinsonian symptoms, but it comes with its own drawbacks, such as postoperative nausea, vomiting, and an increased risk of aspiration pneumonia [7].

Discontinuation of anti-parkinsonian medication for an extended period—exceeding 6 to 12 h—can result in a significant exacerbation of Parkinson’s disease symptoms. The half-life of levodopa is relatively short, approximately 90 min, whereas the half-life of dopamine agonists can vary considerably. Abrupt cessation of medication may lead to a syndrome characterized by hyperpyrexia and parkinsonism, which can be difficult to distinguish from malignant neuroleptic syndrome. This condition is associated with symptoms such as rigidity, hyperpyrexia, dysautonomia, and elevated creatinine levels [8].

Most endoscopic procedures are performed under moderate sedation and analgesia, commonly referred to as “conscious sedation”. Propofol is the primary pharmacological agent used to induce and maintain sedation during upper gastrointestinal endoscopy [9,10].

Procedural sedation and analgesia are fundamental competencies of anesthetists. Recent advancements in patient monitoring and the introduction of novel sedative and analgesic agents have improved both safety and efficacy in this practice [11].

Many anesthetics and adjuvants interact with levodopa. Ketamine and esketamine impact dopaminergic pathways, exacerbating dyskinesias, while commonly used sedatives such as midazolam and thiopental may cause cognitive dysfunction or reduce levodopa efficacy. Opioids, frequently used for pain management, can worsen constipation and sedation, compounding levodopa’s side effects. Dopamine antagonists like metoclopramide can aggravate PD symptoms and should be avoided as antiemetics. Centrally acting analgesics should also be avoided; however, NSAIDs and acetaminophen are generally well tolerated in the postoperative setting [8,12].

The objective of this study is to assess the efficacy of target-controlled infusion (TCI) propofol-based sedation in patients with advanced PD and to develop an internal protocol for managing these patients.

Our primary outcome was to evaluate the real-life effectiveness of this sedation protocol, specifically in terms of procedural success rates.

## 2. Materials and Methods

This study was conducted with the approval of the Ethics Committee of the University of Medicine and Pharmacy, Târgu Mureș (Approval No. 94/19.05.2017). General Data Protection Regulation (GDPR) compliance was ensured, and all data were used exclusively for research purposes.

We conducted a prospective study on patients with advanced Parkinson’s disease who were hospitalized at Târgu Mureș County Emergency Clinical Hospital, Romania, for the implantation of a percutaneous endoscopic gastrojejunostomy (PEG-J) tube. This procedure enables the continuous delivery of levodopa–carbidopa intestinal gel (LCIG) directly into the proximal jejunum.

The inclusion criteria were as follows:

- Advanced stage of Parkinson’s disease—Hoehn and Yahr stages 4 and 5 [13].
- Treatment with levodopa at least four times daily, in combination with dopamine agonists, monoamine oxidase B inhibitors, catechol-O-methyl transferase inhibitors, and/or amantadine.

Patients were hospitalized according to national regulations for titration, initiation of LCIG therapy, dose adjustments, and assessment of treatment efficacy. The anticipated LCIG morning dose and continuous infusion rate were calculated based on published recommendations [14]. To maximize therapeutic benefit, LCIG doses were continually adjusted during the titration period and after the insertion of the PEG-J system. All patients had a nasojejunal tube inserted under endoscopic guidance and were pretested to ensure they would respond to Levodopa treatment.

In this study, the sample size was not determined using a formal statistical calculation. Instead, we included all consecutive patients meeting the inclusion criteria who underwent PEG-J placement between 2011 and 2024 at our institution.

Collected Variables: Demographic data included age, sex, BMI, and ASA score. Procedural data encompassed sedation duration and the total doses of propofol, fentanyl, sufentanil, and midazolam. Clinical outcomes were assessed based on procedural success rate, sedation-related complications, and hospital length of stay.

Patients were monitored for 30 days post-procedure to evaluate treatment outcomes.

Sedation was induced and maintained with propofol (Fresenius, Austria) via target-controlled infusion (TCI) using a Diprifusor TCI pump (Fresenius Orchestra), based on the Marsh model. Anesthesia was supplemented with the co-administration of benzodiazepines and opioids (Sufentanil or Fentanyl) on demand; no muscle relaxants were used.

### 2.1. Adapted TCI Protocol

1. Pre-induction phase
  - Patients were advised to consume clear fluids (e.g., water, pulp-free juice, tea, or coffee without milk) until two hours before surgery, while a fasting period of six hours was required for solid food.
2. Monitoring
  - Patient monitoring during TCI followed standard anesthesia and recovery guidelines established by the Association of Anesthetists.
  - Hemodynamic monitoring included ECG, blood pressure (BP), and oxygen saturation (SpO_2_).
  - Secure IV access and preload fluids were ensured.
  - Preoxygenation was achieved by administering 100% oxygen for three minutes before induction.
3. Drugs for induction
  - Propofol 1% was used for induction. We use a slow maximum/bolus infusion rate (600 mL/h) for TCI to avoid hypotension.
  - Closed-loop control was used for TCI. The initial propofol target concentration was set at 2 µg/mL, then gradually increased until the patient became unresponsive. The achieved target concentration was confirmed on the TCI device to enable continuous monitoring during the awakening period after infusion cessation.
  - In some cases, benzodiazepines and/or short-acting opioids (fentanyl/sufentanil) were administered as needed.
4. Maintenance and procedure
  - All patients were premedicated with benzodiazepines—Midazolam (Hypericum, Romania) at a dose of 0.3 mg/kg.
  - Propofol target concentration: 2–3 µg/mL (Table 1).
  - Oxygen administration was provided via a nasal cannula at a flow rate of 3–5 liters per minute, while spontaneous respiration was maintained.
  - All patients received 1% lidocaine (Zentiva, Bucharest) as a local anesthetic, administered by the gastroenterologist. The lidocaine was delivered by creating a subcutaneous bleb and infiltrating along the axis of the proposed PEG insertion, reaching the stomach’s depth.
5. Emergence
  - Propofol infusion was discontinued at the conclusion of surgery.
  - The Aldrete score was monitored until the patient achieved a score greater than 9 points, ensuring safe discharge from the post-anesthesia care unit (PACU).
6. Postoperative management
  - Postoperative pain management: Non-opioid analgesia administered acetaminophen (1 g IV every 8 h as needed) and NSAIDs (ketoprofen 100 mg IV every 12 h as needed).
  - Patients were monitored for postoperative pain using a numeric scale (0 to 10) according to our hospital protocol, and analgesia is recommended at pain levels higher than 3.
  - Antiemetics: Dopaminergic receptor antagonists were avoided. Instead, serotonin receptor antagonists such as ondansetron or granisetron were used on demand.

### 2.2. Statistical Analysis

All collected data were recorded in a database and analyzed using Microsoft Excel and MedCalc. Descriptive statistics (mean, median, and standard deviation) were calculated for continuous variables.

## 3. Results

Between 2011 and 2024, a total of 169 consecutive patients diagnosed with advanced PD were admitted to the Emergency County Clinical Hospital in Târgu Mureș, Romania, for PEG-J insertion.

Among them, 88 patients (52%) were men, and 81 patients (48%) were women. The median age was 65 years (Table 2).

Among all patients, 11.2% (n = 19) received short-acting opioid boluses during the procedure—fentanyl (Kalcex, Riga, Latvia) (0.05–0.15 mg) in 12 patients and sufentanil (Medochemie Ltd., Limassol, Cyprus) (5–10 µg) in 17 patients. Opioids were used for patients who remained responsive during the procedure despite high plasma propofol concentrations (above 4 µg/mL).

No severe anesthetic or procedural complications occurred. Hypotension, defined as MAP < 30% drop from the initial value, was observed in 13 patients (7.7%), all of whom experienced transient episodes that were effectively managed with IV fluid administration, without the need for vasopressor support. Only one patient required a single intravenous bolus dose of 10 mg ephedrine (Zentiva, Czech Republic).

Oxygen desaturation below 92% was recorded in one patient, but it was self-limiting and did not necessitate airway intervention or additional respiratory support. All patients maintained adequate spontaneous ventilation throughout the procedure, ensuring overall procedural safety.

No deaths were reported within 30 days post-procedure.

## 4. Discussion

Patients diagnosed with Parkinson’s disease (PD), typically older individuals with multiple comorbidities, often require continuous intestinal infusion of carbidopa/levodopa gel via PEG-J tube placement to improve motor function and quality of life. The median age in our study was 65 years, with most participants having an ASA score of 3, indicating significant systemic disease without an immediate threat to life [15].

This study supports the efficacy of TCI with propofol for sedation management during PEG-J placement in patients with advanced PD. The protocol showed a high procedural success rate, underscoring its feasibility and safety within a complex patient population.

Patients with PD may experience acute deterioration during the perioperative period due to concurrent illnesses and medication alterations, leading to an exacerbation of both motor and non-motor symptoms. These risks are heightened in the advanced disease, where higher doses of levodopa are required. Potential complications include severe off-period symptoms such as orthostatic hypotension, acute psychosis, delirium, recurrent motor fluctuations, significant dyskinesia, and akinetic crisis [6].

Additionally, medications such as levodopa and dopaminergic agonists can exacerbate hypotension, necessitating careful monitoring and adjustments to concomitant hypotensive agents [12]. Hypotension is defined as MAP < 30% drop from the initial value, and 13 patients (7.7%) experienced transient hypotension managed with IV fluids. None required vasopressor support.

Gravina et al. conducted a retrospective study involving 65 PEG-J procedures in PD patients, utilizing a comparable TCI-propofol protocol supplemented with atropine and midazolam. Their study, however, was retrospective in nature, whereas ours is a prospective study including a larger patient cohort (169 patients). Additionally, our protocol did not include atropine and focused on the real-world effectiveness of sedation management without the need for supplementary anticholinergic agents. Their results demonstrated a 98% procedural success rate, which aligns with our findings and further reinforces the safety and efficacy of TCI-propofol sedation [9]. The findings of our study align with recent research on sedation protocols for patients with PD. Notably, many of these studies involved smaller patient cohorts compared to our investigation, strengthening the statistical reliability and generalizability of our findings.

Similarly, Nan Jiang et al. evaluated TCI-propofol sedation in 68 PD patients undergoing deep brain stimulation, demonstrating its effectiveness in achieving balanced sedation while reducing hemodynamic fluctuations, a critical factor for PD patients prone to autonomic instability; consistent with their findings, our study further supports the advantages of TCI-propofol over weight-adjusted propofol dosing (mg/kg) by ensuring precise titration, reducing the risk of over-sedation or insufficient sedation, and enhancing hemodynamic stability, thereby minimizing hypotension, bradycardia, and respiratory complications, which are particularly relevant in elderly patients with autonomic dysfunction [16]. Dexmedetomidine has been explored as an alternative sedative agent in PD due to its minimal respiratory depression and neuroprotective effects; however, studies have shown that its prolonged onset time and risk of severe bradycardia limit its applicability for short procedures like PEG-J placement [7].

The perioperative management of PD should address two key considerations:

1. Disease-specific challenges;
2. The pharmacological treatments employed.

Advanced PD presents significant perioperative hurdles, including the exacerbation of motor symptoms due to pharmacotherapy alterations and non-motor complications, such as autonomic dysfunction [8]. Our approach—maintaining anti-Parkinsonian medication as close to surgery as possible—was instrumental in mitigating these risks. The use of propofol via TCI provided precise control over sedation depth, reducing the risk of oversedation and associated complications such as aspiration pneumonia.

The integration of PEG-J placement with TCI sedation has important clinical implications. Continuous jejunal delivery of levodopa–carbidopa significantly enhances motor function and quality of life in patients with advanced PD [17]. Our protocol’s focus on safety and efficacy supports its broader adoption in specialized centers. Additionally, insights from this study can inform updates to perioperative protocols, emphasizing the judicious use of sedatives and close monitoring of both motor and non-motor symptoms during the perioperative period.

### Limitations

While the findings of this study are promising, several limitations should be acknowledged. The single-center design may limit generalizability, as results may not fully reflect broader clinical settings. Additionally, the absence of a control group prevents direct comparisons with alternative sedation techniques or anesthesia protocols. This study did not include objective monitoring of sedation depth and analgesia (e.g., BIS or entropy), as it was not part of the institutional practice at the time. Future research should include larger, multicenter cohorts and employ comparative methodologies to enhance external validity and address these limitations.

## 5. Conclusions

This study highlights the feasibility and clinical applicability of a TCI propofol protocol for PEG-J placement in patients with advanced PD (stages 4 and 5). While no deaths were recorded within the 30-day follow-up, the sample size is insufficient to draw definitive conclusions regarding long-term safety. The absence of mortality in this cohort suggests that this sedation approach is a viable option for this patient population; however, further studies with larger cohorts and comparative designs are necessary to establish its safety and effectiveness conclusively. By addressing the specific perioperative challenges of this population, the protocol may contribute to improved procedural outcomes and enhanced patient care. Continued research is essential to refine these protocols and validate findings across diverse clinical settings.

## Figures and Tables

**Table 1:** Anesthesia and surgery data.

	Median	Mean	Minimum	Maximum	Standard Derivation
LOS in hospital (days)	9	10.12	1	38	4.14
Length of anesthesia (minutes)	20	21.63	10	75	9.23
Propofol plasma concentrations (μg/mL)	3	2.99	2	6	0.89
Cumulative propofol (mg)	246	265.89	30	1012.5	137.637
Cumulative sufentanil (µg)	10	10	5	10	2.61
Cumulative fentanyl (mg)	0.075	0.09	0.05	0.15	0.045
Cumulative midazolam (mg)	1	1.68	0.5	5	1.3
ASA score (points)	3	3	2	4	0.046

LOS—length of stay; ASA—American Society of Anesthesiologists Physical Status Classification.

**Table 2:** Demographic data.

	Median	Mean	Minimum	Maximum	Standard Derivation
Age (years)	65	64	39	83	8.3
Height (cm)	168	167.33	145	196	8.9
Weight (kg)	76	75.69	35	123	48.69
BMI (kg/m^2^)	26.9	26.9	14.9	40.89	16.81
Initial MAP (mmHg)	102	81.92	75	133	20.17
Lowest MAP (mmHg)	85	81.92	55	112	18.15
Initial SpO_2_ (%)	98	97.95	92	100	1.87
Lowest SpO_2_ (%)	98	97.89	90	100	1.9

BMI—body mass index, MAP—mean arterial pressure.

## Data Availability

The data used for this study can be found in the database of the Târgu Mures, County Emergency Clinical Hospital, Romania.
